# Supplementary material for: Environmental remodeling of human gut microbiota and antibiotic resistome in livestock farms
Source: Nat Commun. 2020 Mar 18;11:1427. doi: 10.1038/s41467-020-15222-y (PMC7080799; doi:10.1038/s41467-020-15222-y)
Supplement: Supplementary file 1 — Supplementary Information [file 41467_2020_15222_MOESM1_ESM.pdf]

Supplementary Information

Environmental remodeling of human gut microbiota and antibiotic resistome in livestock farms

Jian Sun[1,2]*, Xiao-Ping Liao[1,2]*, Alaric W. D'Souza[3]*, Manish Boolchandani[3]*, Sheng-Hui Li[1,4]*, Ke Cheng[1,2], José Luis Martínez[5], Liang Li[1,2], You-Jun Feng[1,2,] Liang-Xing Fang[1,2], Ting Huang[1,2], Jing Xia[1,2], Yang Yu[1,2], Yu-Feng Zhou[1,2], Yong-Xue Sun[1,2,6], Xian-Bo Deng[2], Zhen-Ling Zeng[1,2,6], Hong-Xia Jiang[1,2,6], Bing-Hu Fang[1,2,6], You-Zhi Tang[1,2,6], Xin-Lei Lian[1,2], Rong-Min Zhang[1,2], Zhi-Wei Fang[4], Qiu-Long Yan[4], Gautam Dantas[3,7,8,9#], Ya-Hong Liu[1,2,6#]

[1] National Risk Assessment Laboratory for Antimicrobial Resistance of Animal Original Bacteria, South China Agricultural University, Guangzhou, Guangdong, China
[2] Guangdong Laboratory for Lingnan Modern Agriculture, Guangzhou, Guangdong, China
[3] The Edison Family Center for Genome Sciences and Systems Biology, Washington University in St. Louis School of Medicine, St. Louis, MO, USA
[4] Shenzhen Puensum Genetech Institute, Shenzhen, Guangdong, China
[5] Centro Nacional de Biotecnología, Consejo Superior de Investigaciones Científicas, Calle Darwin, Madrid, Spain
[6] Guangdong Provincial Key Laboratory of Veterinary Pharmaceutics Development and Safety Evaluation, South China Agricultural University, Guangzhou, Guangdong, China
[7] Department of Pathology & Immunology, Washington University in St. Louis School of Medicine, St. Louis, MO, USA
[8] Department of Biomedical Engineering, Washington University in St. Louis, St. Louis, MO, USA
[9] Department of Molecular Microbiology, Washington University in St. Louis School of Medicine, St. Louis, MO, USA

* These authors contributed equally to this work.

[#] e-mail: dantas@wustl.edu; lyh@scau.edu.cn

Supplementary Notes

Summary information about the questionnaire

Fourteen students and 14 farm workers were asked to participate in this study and all of them finished the questionnaire (Supplementary Questionnaire). According to the survey, we found that although the geographical position and the breeding scale are different, the management level such as hygienic condition and labor management model are quite consistent in the three individual farms (Supplementary Fig. 1a). In short, the three farms are close-ended management mode and had opened at least 6 years. The pig farms were divided into production areas and living areas. The workers work in the production area, and came back to the living area after get off work, where providing the free accommodation and catering services. The workers have a strict schedule and are only allowed one day off per week. As for the antibiotic and metal prescription, in the three pig farms, were also highly similar including penicillin, amoxicillin, gentamicin, florfenicol, enrofloxacin, sulfonamides and so on. This profession is male dominated in China, and consequently all of the volunteers in this study are male (Supplementary Table 1). The average age of the students is only 24 years old, while the farm workers are as old as 44 years. Compared to the short stay in the farm (3 months) for students, ten out of the fourteen workers have engaged in the pig industry for more than five years. Among them, five have more than 10 years of experience, and the most experienced had been working for 18 years. Moreover, all tested workers had worked in the current pig farms for more than one year. The workers were healthy, except for one that had chronic rhinitis and was prescribed an antibiotic through outpatient care 6 months ago. One other worker caught a cold but without any antibiotic treatment. In contrast, 6 of the 14 students had experience at a hospital within the last one year (1 inpatient, 4 outpatients, and 1 visitor). l However, only 4 of them took antibiotics within the last 6 months. The workers and students had similar diets. Almost all of the participants have meat centered diets, and they showed great interest in pork, chicken, and fish for every meal. The questionnaire was originally in Chinese, but it is translated into English for this publication.

Analysis of the metagenomic data from previous Chinese project

We extracted the metagenomic data from 368 adult fecal samples of the previous Chinese project[1], of that, two samples were excluded for unusual high faction of *Escherichia coli* (88.1% and 94.4% of relative abundance). All of these adults were recruited from the urban residents of two cities of the Guangdong province: Guangzhou and Shenzhen. The microbial composition of the Chinese samples were obtained based taxonomic annotation of their existing gene catalogue (4.23 million non-redundant genes[1]) via the same pipeline used in our study (Online Methods).

Overview of the swine farm environmental microbiome and resistome

To characterize the environmental microbiomes and antibiotic resistomes in three swine farms, we performed whole-metagenome shotgun sequencing on four typical environments (dust, swine feces, sewage and soil) for each swine farm. We collected the same amount of raw materials in 3-5 sampling spots for each environment of each swine farm, after that, totaling 12 pooled environmental samples were analyzed. After removing the low-quality and/or host-derived data (Online Methods), we generated a total of 133.2 Gb (11.1 Gb per sample on average) high-quality sequencing reads in these samples (Supplementary Table 5). *De novo* assembly generated a total of 12,877,381 contigs (total length 9,052,706,533 bp, with average N50 length 848 bp). Notably, comparing that average 88.7% reads were assembled to contigs in the human fecal samples of students and swine farm workers (Supplementary Table 4), only average 60.9% reads (average 63.2% in dust samples, 76.7% in swine feces samples, 65.3% in sewage samples and 38.5% in soil samples) were enabled to map to the assembled contig set of each environmental sample (Supplementary Table 5), indicating that a large number of genomic sequences in the environmental sample were still unexplored, especially for the soil samples. We identified a total of 15,835,043 genes in these contigs (representing 76.8% of the contig sequences), and compiled a non-redundant catalogue of 11,374,480 genes using CD-HIT[2]. Specially, this gene catalogue is much larger than the human gut gene catalogue based on 45 fecal samples in our study (containing 3.34 million genes), and even larger than the current largest human gut gene

catalogue based on 1,267 worldwide fecal samples and 511 human gut-associated microbes (containing 9.89 million genes)[3]. Based on the extensive environmental gene catalogue, we first investigated the completeness of the microbiota in the three swine farms. Rarefaction analysis (Supplementary Fig. 6b) showed that the observed number of genes approached saturation in most samples under the current number of sequencing reads. Only 8.2% genes (0.9/11.4 million) in the environmental gene catalogue were shared in at least two habitats (Supplementary Fig. 6a), especially, only 0.2% (~20,000) genes were existed in all four habitats, suggesting that the genes exchange between different habitats is not widespread.

We then characterized the phylogenetic composition across the environmental samples at the phylum and genus levels, based on taxonomic assignment of the non-redundant genes (Online Methods). At the phylum level, the samples were dominated with Proteobacteria (average relative abundance, 37%), Firmicutes (26%), Bacteroidetes (15%), Actinobacteria (13%), Euryarchaeota (4%) and Spirochaetes (2%) (Supplementary Fig. 7a), while the abundance of other phyla were less than 1%. At the genus level, the samples were composed by a variety of genera (see Supplementary Fig. 7b for the top 20 genera in all samples). In detail, the most dominate genera were *Gardnerella* (20%) and *Brachybacterium* (16%) in the dust microbiomes, *Escherichia* (19%) and *Serratia* (13%) in the swine fecal microbiomes, *Methanosaeta* (18%) and *Serratia* (13%) in the sewage microbiomes, and *Serratia* (39%) and *Pseudomonas* (12%) in the soil microbiomes.

We identified a total of 2,331 antibiotic resistance genes from the non-redundant gene catalogue of the environmental microbiomes. The AR genes were more widespread in different habitats than the other genes in the whole gene catalogue – for example, 19.5% AR genes were shared in at least two habitats (Supplementary Fig. 7c), and 7.2% (174) AR genes were existed in all four habitats. This finding confirmed the previous studies[4, 5] showing a highly cosmopolitan antibiotic resistance proteins across human, animal and environmental resistomes. Averagely, 39% AR gene in the environmental samples were novel to known proteins (<90% amino acid identity to any protein in NCBI-NR, Supplementary Fig. 7d), which is significantly higher than the human gut resistomes (P<0.001), suggesting that a diverse, unexplored antibiotic resistance gene pool is maintained in the natural swine farm environment.

Supplementary Table 1 Summary information about the questionnaire.

	Student (n = 14)	Worker (n = 14)
Gender (F/M)	0/14	0/14
Age, years	24 ± 1	44 ± 8
Engaging period	3 ± 0.5 months	9 ± 5 years
Living on swin farm (Y/N)	14/0	14/0
Working period (per week), hours	48-56	48-56
Eating meat (per week)	100%	100%
Dietary habits		
pork	14 (100%)	14 (100%)
chicken	14 (100%)	14 (100%)
fish	14 (100%)	14 (100%)
Physical Status within the last year		
healthy	10 (71%)	12 (86%)
as outpatient	3 (21%)	2 (14%)
in hospital	1 (8%)	0 (0%)
Frequency of treating with antibiotics within the last 6 months		
none	8 (57%)	12 (86%)
1-5	5 (36%)	2 (14%)
>5	1 (7%)	0 (0%)

Supplementary Table 2 Primers used in this study.

Gene	Primer (5’ - 3’)	Reference
bla$_{CTX-M}$	F: TTTGCGATGTGCAGTACCAG<br>R: CGATATCGTTGGTGGTGCC	[6]
fosA3	F: GCGTCAAGCCTGGCATTT<br>R: GCCGTCAGGGTCGAGAAA	[7]
Variable region	F: CTGGCGTAACCCTTCCGAT<br>R: TTCATCACCGCGATAAAGCA	This study

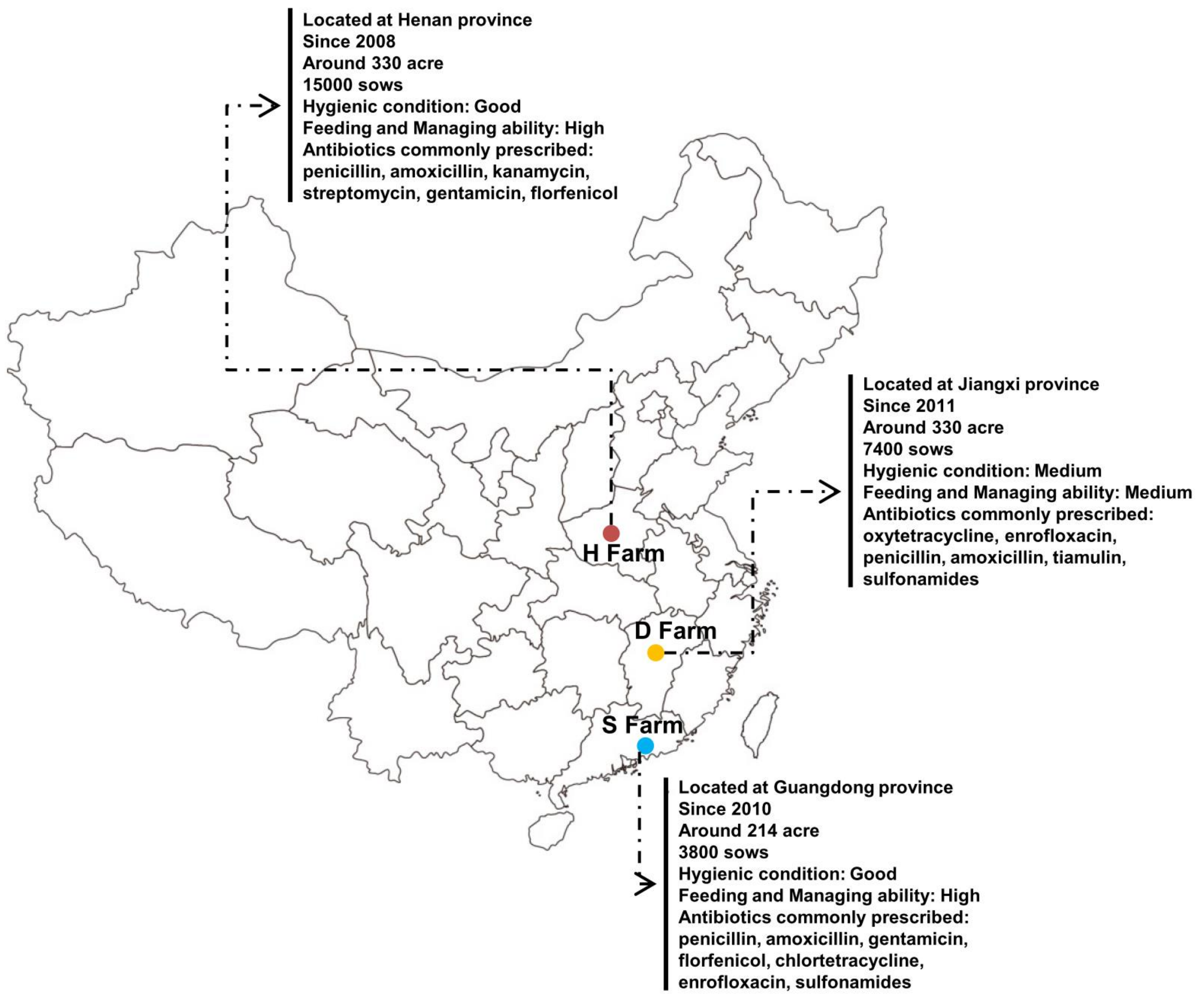

Supplementary Figure 1 | Location and overview of study sites in China. Red dot, Farm H; yellow dot, Farm D; blue dot, Farm S.

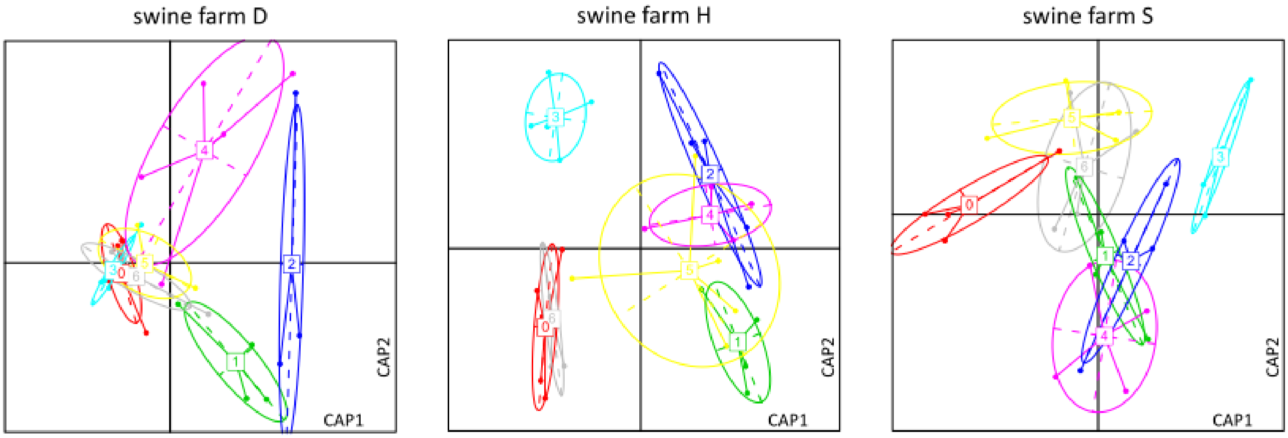

Supplementary Figure 2 | DbRDA analysis of the Bray-Curtis distances between gut microbiota in samples on three swine farms. The first and second principal components are shown. The nodes represent the samples, the lines connect samples obtained at the same time points, and the colored circles indicate the samples near the center of gravity for each time point. Source data are provided in the Source Data file.

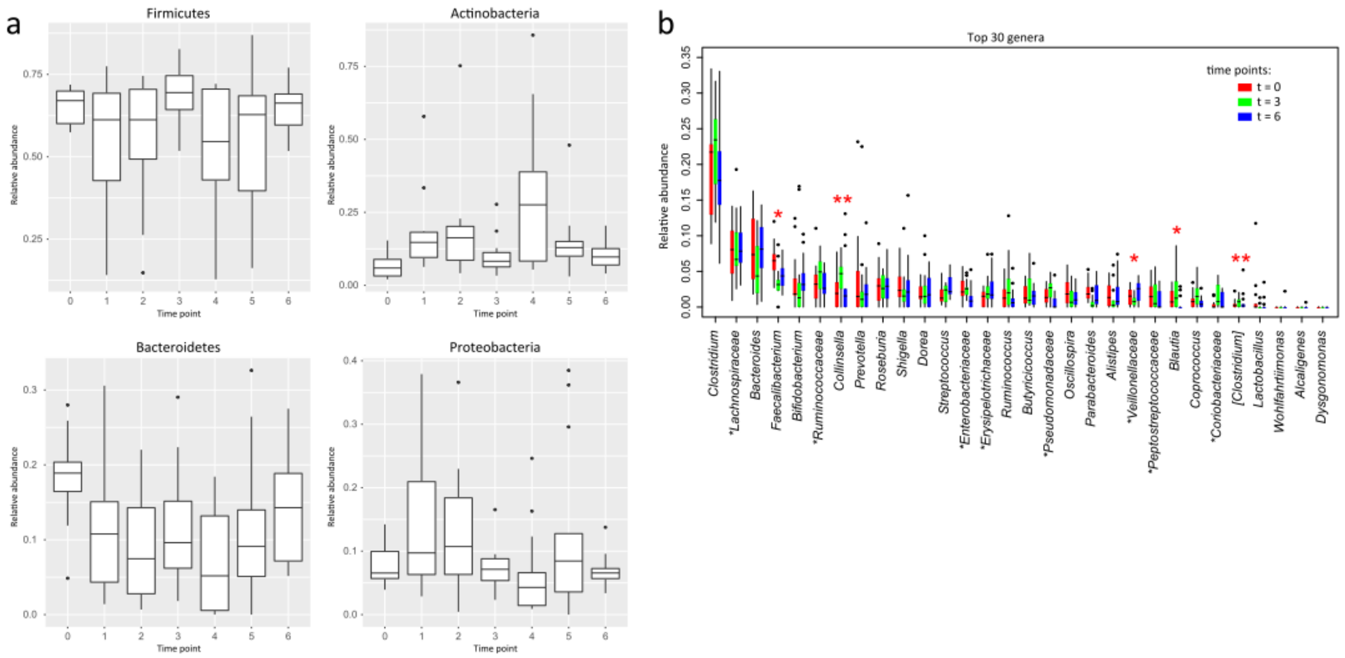

Supplementary Figure 3 | Alteration of gut microbial composition following environmental conversion. (a) Change in the relative abundance of the top 4 dominant phyla. (b) Relative abundance of the top 30 dominant genera in students' gut microbiota at time points T0, T3 and T6. The box and scatter plots in a and b show the distribution of the samples (the boxes show medians/quartiles; the error bars extend to the most extreme value within 1.5 interquartile ranges). For b *$P < 0.05$, **$P < 0.01$, paired Student's t-test. Source data are provided in the Source Data file.

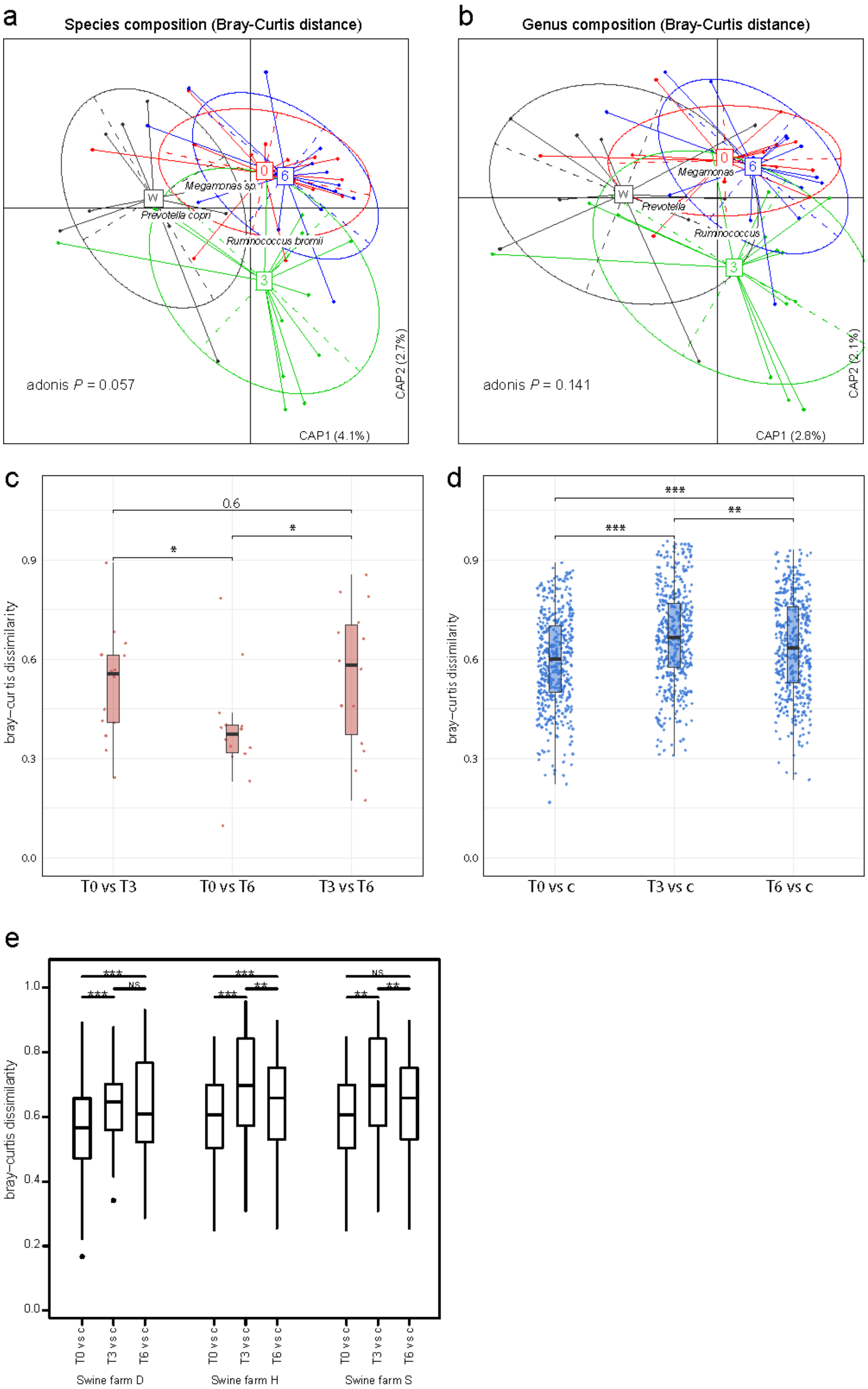

Supplementary Figure 4 | Alteration of gut microbiota as revealed by whole-metagenome data. (a-b) Distance-based redundancy analysis (dbRDA) of the microbial species- (a) and genus-level (b) composition of students' samples at time points T0 (red), T3 (green) and T6 (blue), and workers' samples. DbRDA plots are shown the first two principal components. Lines connect samples from the same time point, and coloured circles indicate the samples near the center of gravity for each time point. (c) Bray-Curtis dissimilarity between student samples at three collection times. Each point is a pairwise comparison between two samples from an individual. (d) Bray-Curtis dissimilarity between student times and control samples. Each point is a pairwise comparison between a student sample and a control sample. (e) Bray-Curtis dissimilarity between student times and controls samples, students are separated into three farms of geographical location differences. **P* < 0.05, ***P* < 0.01, *** P < 0.001 Wilcoxon test. Source data are provided in the Source Data file.

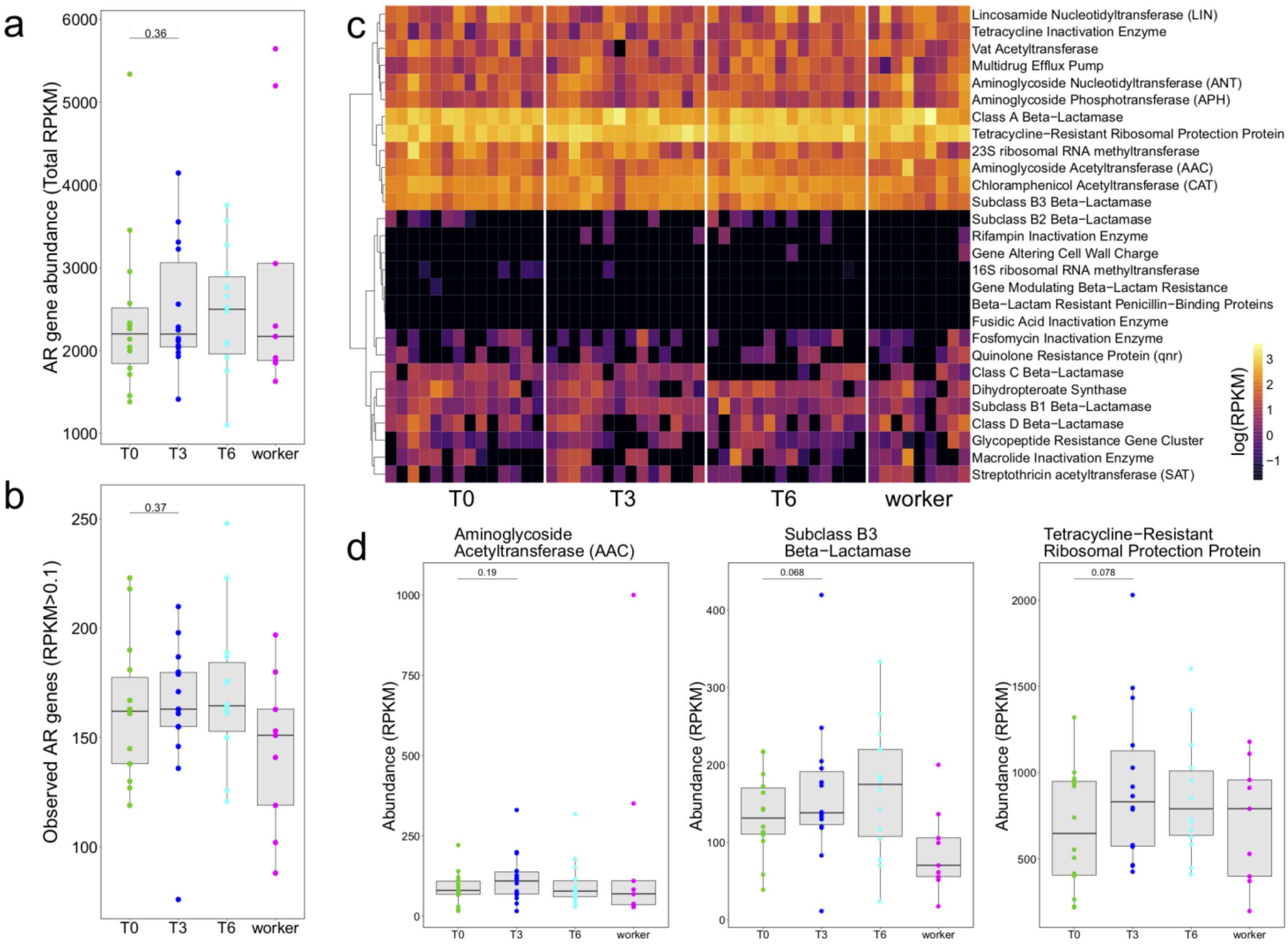

Supplementary Figure 5 | Changes in the number and abundance of AR genes in the antibiotic resistome during the swine farm residence period. (a) Observed total AR gene abundance in RPKM and (b) Observed number of unique AR genes with RPKM>0.1 in the students' gut microbiota at time points T0, T3 and T6, and in the workers' gut microbiota. Boxes show medians/quartiles; error bars extend to the most extreme values within 1.5 interquartile ranges. (c) Heatmap showing the composition of AR types in the students' and workers' gut microbiota. (d) Box plot showing the abundance of three AR types that significantly increased during the students' residence at the swine farm. Boxes show medians/quartiles; error bars extend to the most extreme values within 1.5 interquartile ranges. P-values are shown on the respective plots with lines indicating the compared groups. P-values are multiple hypothesis test corrected using Benjamini-Hochberg (FDR) correction. Source data are provided in the Source Data file.

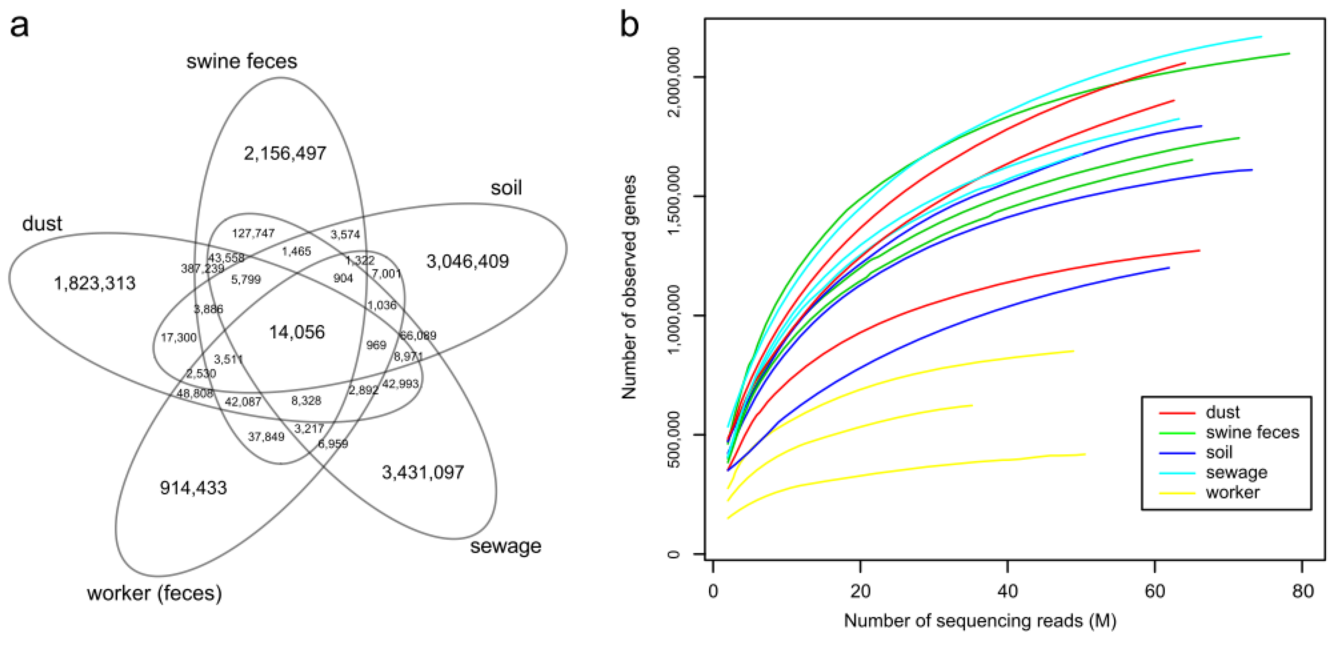

Supplementary Figure 6 | Summary of microbial gene content in the swine farm ecosystem. (a) Comparison of the non-redundant genes found in the swine farm environmental and worker fecal samples. (b) Rarefaction of genes observed in environmental and worker fecal samples. The number of genes in each sample was calculated after 30 random samplings with replacement in different numbers of sequencing reads.

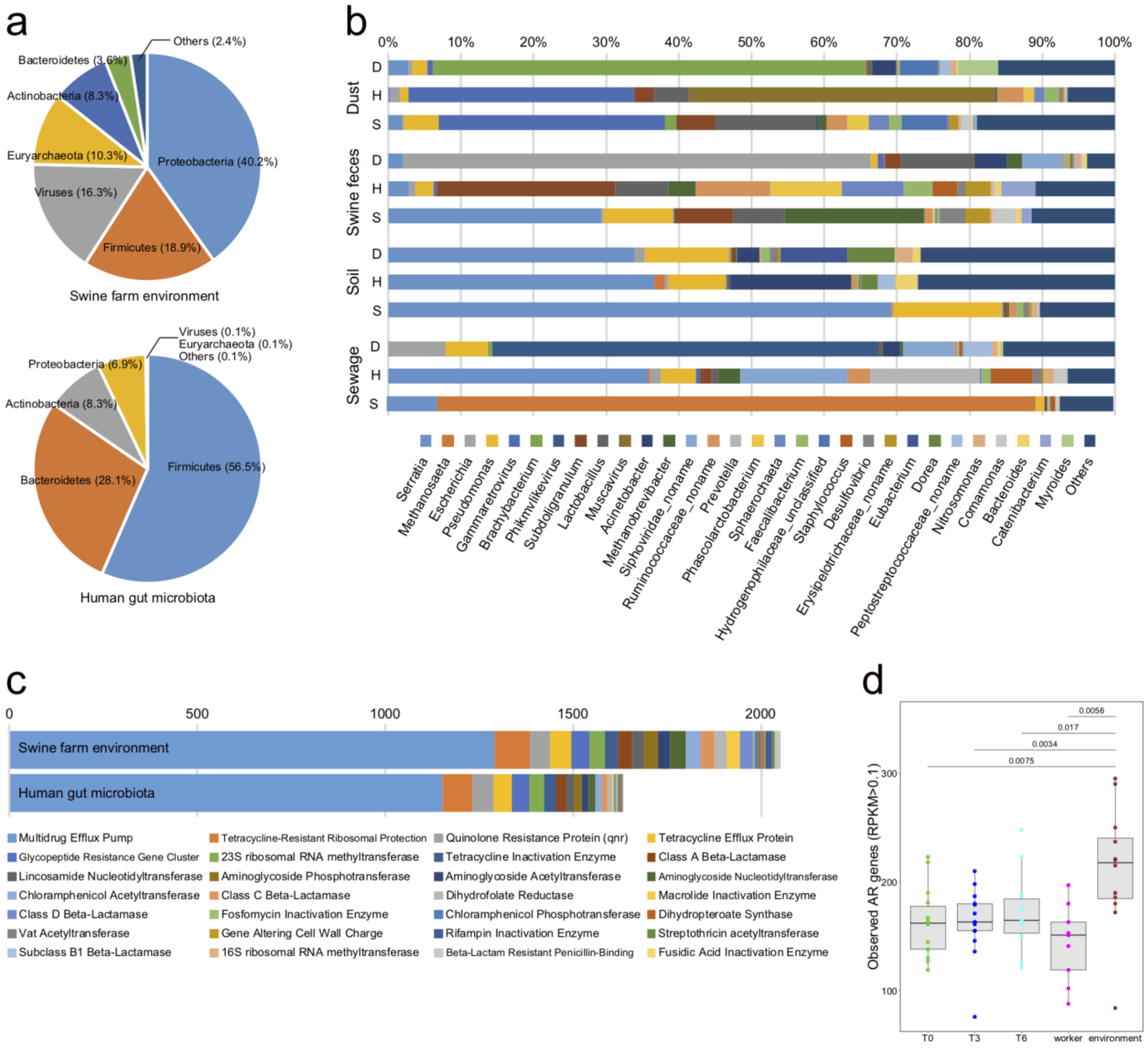

Supplementary Figure 7 | Overview of the environmental microbiome and antibiotic resistome. (a) Comparison of the phylum-level microbial composition of the swine farm environments and the human feces. (b) Genus-level microbial composition of the swine farm environmental samples. (c) Comparison of the AR genes in the swine farm environmental and worker fecal samples. (d) Observed number of AR genes in the human gut microbiota and the environmental samples. Boxes show medians/quartiles; error bars extend to the most extreme values within 1.5 interquartile ranges. P-values are shown on the plot with lines indicating the compared groups. P-values are multiple hypothesis test corrected using Benjamini-Hochberg (FDR) correction. Source data are provided in the Source Data file.

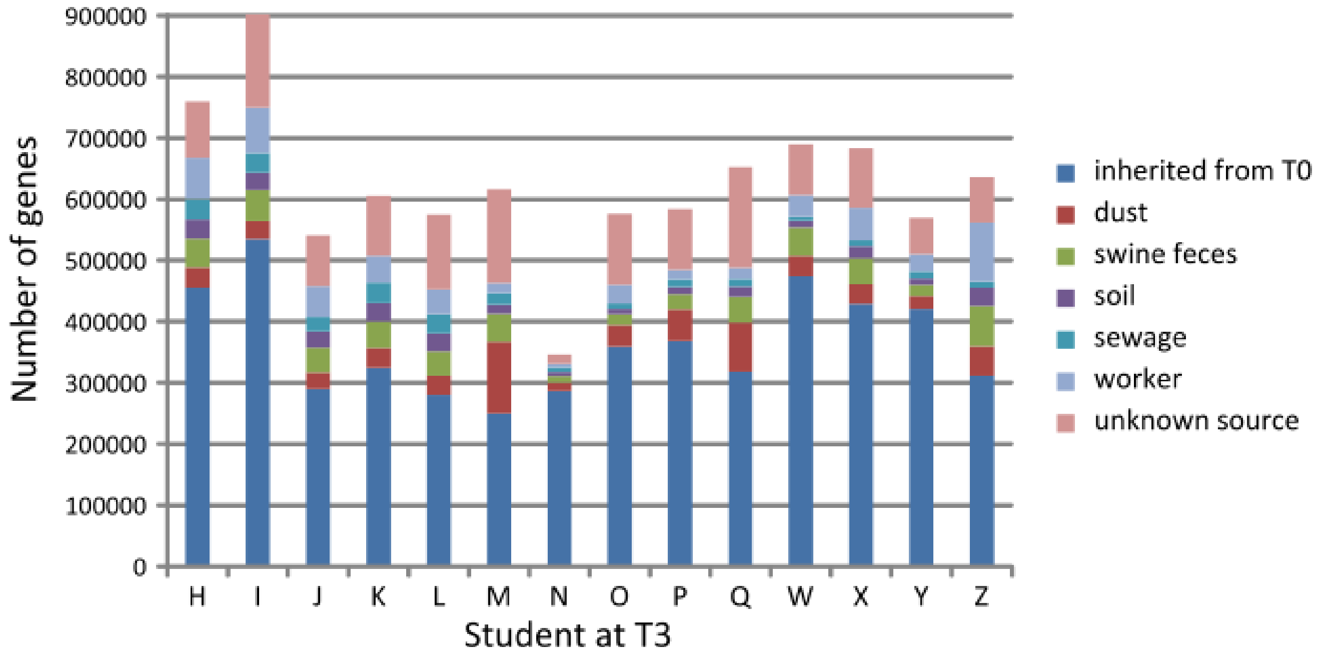

Supplementary Figure 8 | Source of genes found in the students' swine farm-stay microbiomes. Genes inherited from the original gut microbiome (T0) and genes that co-existed with the environmental and swine farm worker samples are shown. Source data are provided in the Source Data file.

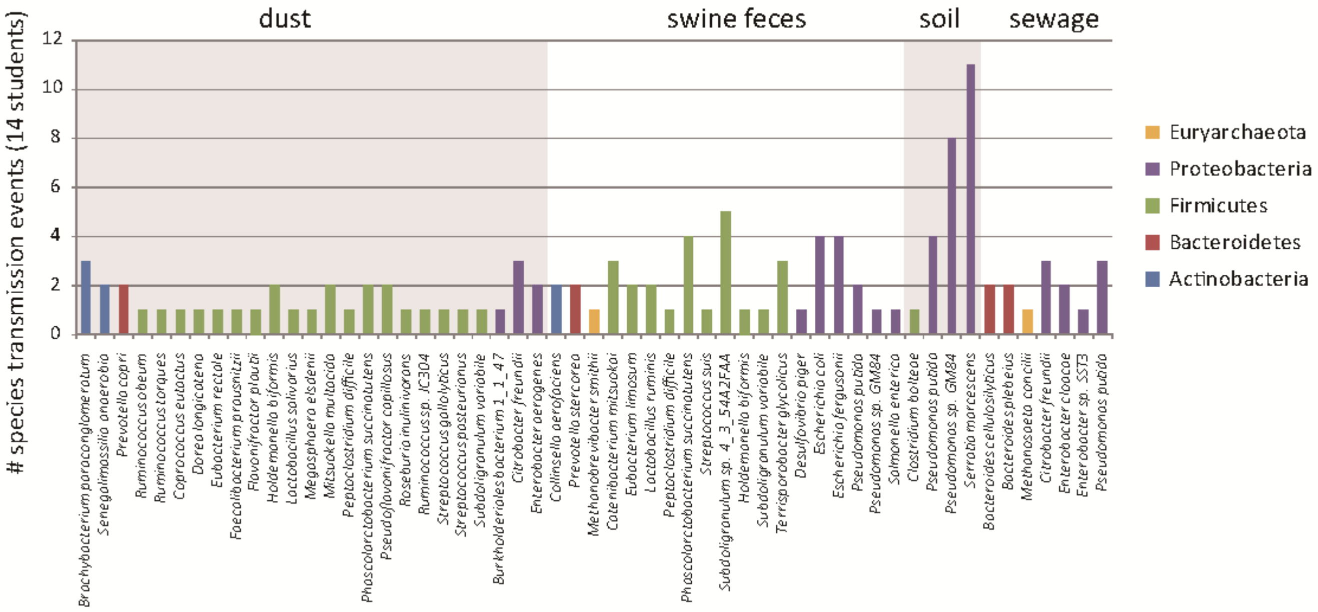

Supplementary Figure 9 | Detailed information on species transmission events. The number of species transmission events observed in each of 14 students is shown; different colors indicate the phylum taxonomic assignments of the species. Source data are provided in the Source Data file.

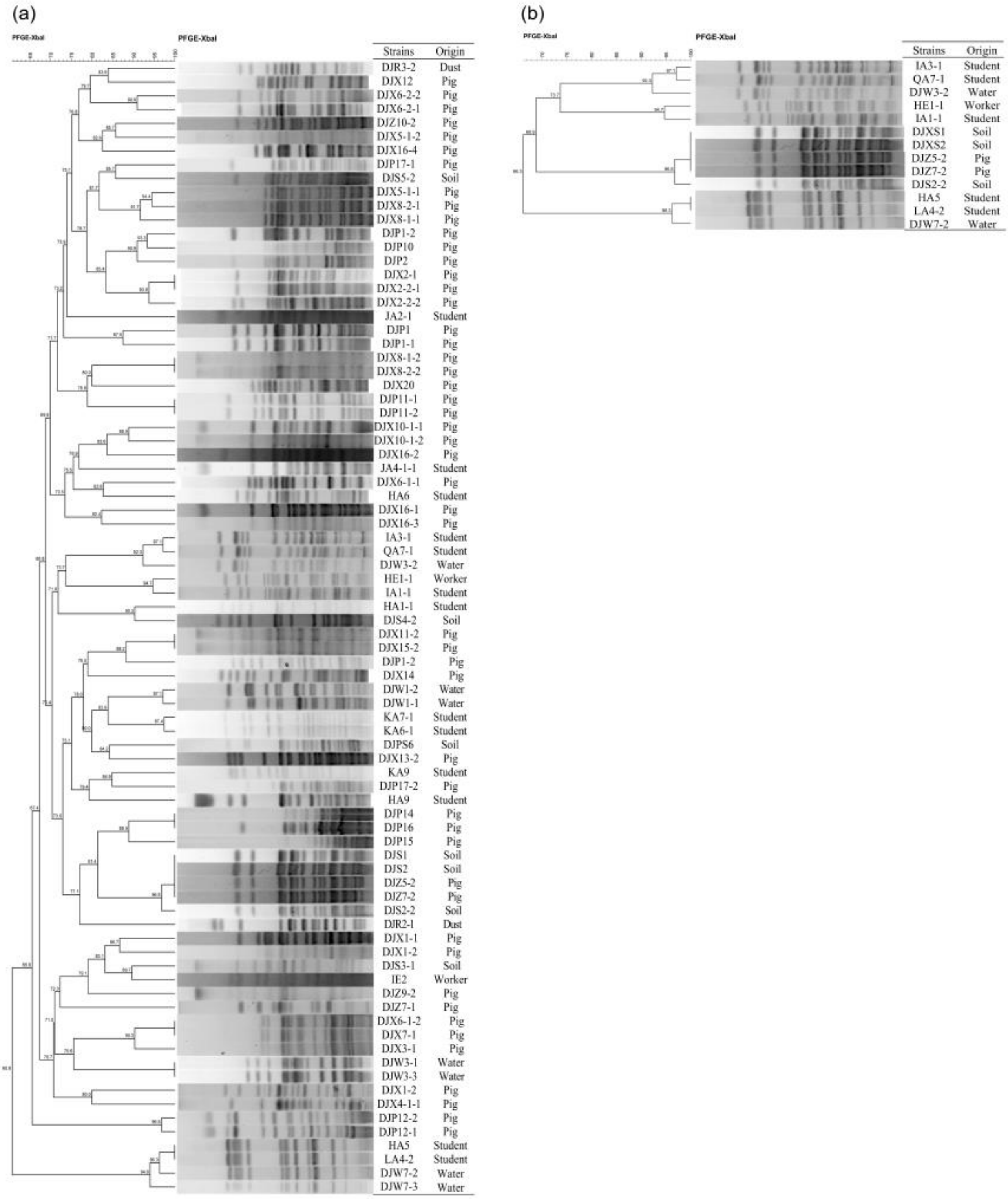

Supplementary Figure 10| Dendrogram illustrates the genetic relatedness of *E. coli* strains isolates by pulsed-field gel electrophoresis (PFGE). (a) The genetic relatedness of 82 *E. coli* strains isolates randomly selected from one pig farm. (b) The genetic relatedness of 13 *E. coli* strains showing clonal spread.

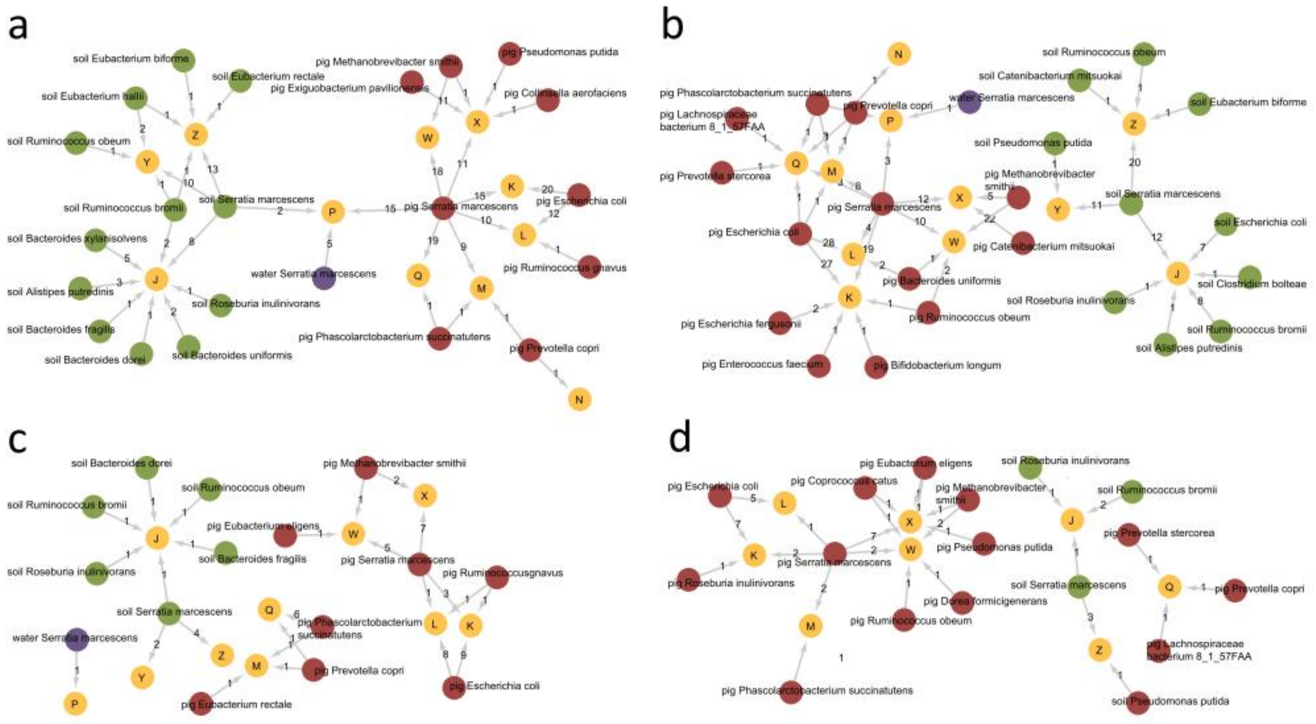

Supplementary Figure 11 | Transfer of bacterial functional genes along with species transmission. Co-transfers of antibiotic resistance genes (a), virulence factors (b), and antibacterial biocide (c) and metal resistance genes (d) via the species transmission networks are shown. The larger nodes depict the students with their IDs displayed in the center. The smaller nodes depict the transmitted species; the different colors indicate different environmental types, and nodes representing high-frequency (>3) species are indicated. The connecting arrows represent transmission events; the numbers within the arrows indicate the number of transferred functional genes. Source data are provided in the Source Data file.

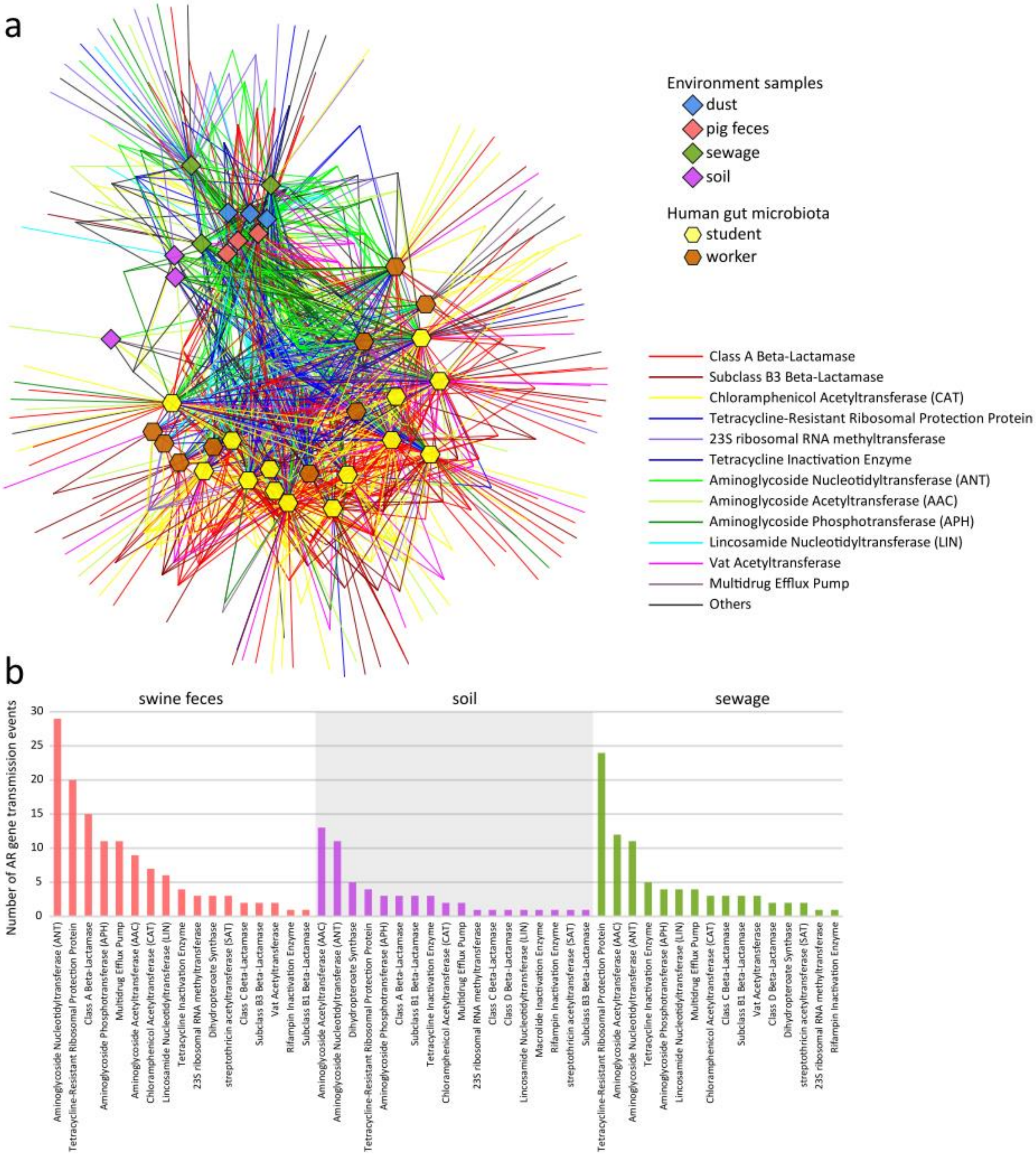

Supplementary Figure 12 | Antibiotic resistance networks and AR gene transmission between human gut and environmental microbiota. (a) Sharing network of AR genes among swine farm environmental and human fecal samples. Lines represent unique AR genes found in at least one sample; the predicted resistance mechanisms are indicated by different colors. And the lines connecting the samples with the AR genes represent ShortBRED hits with an RPKM of ≥10. The large nodes represent individual human gut (hexagon) or environmental (rhombus) samples. (b) Number of antibiotic resistance genes transmitted from the environments to the students' gut, as identified by SourceTracker. Source data are provided in the Source Data file.

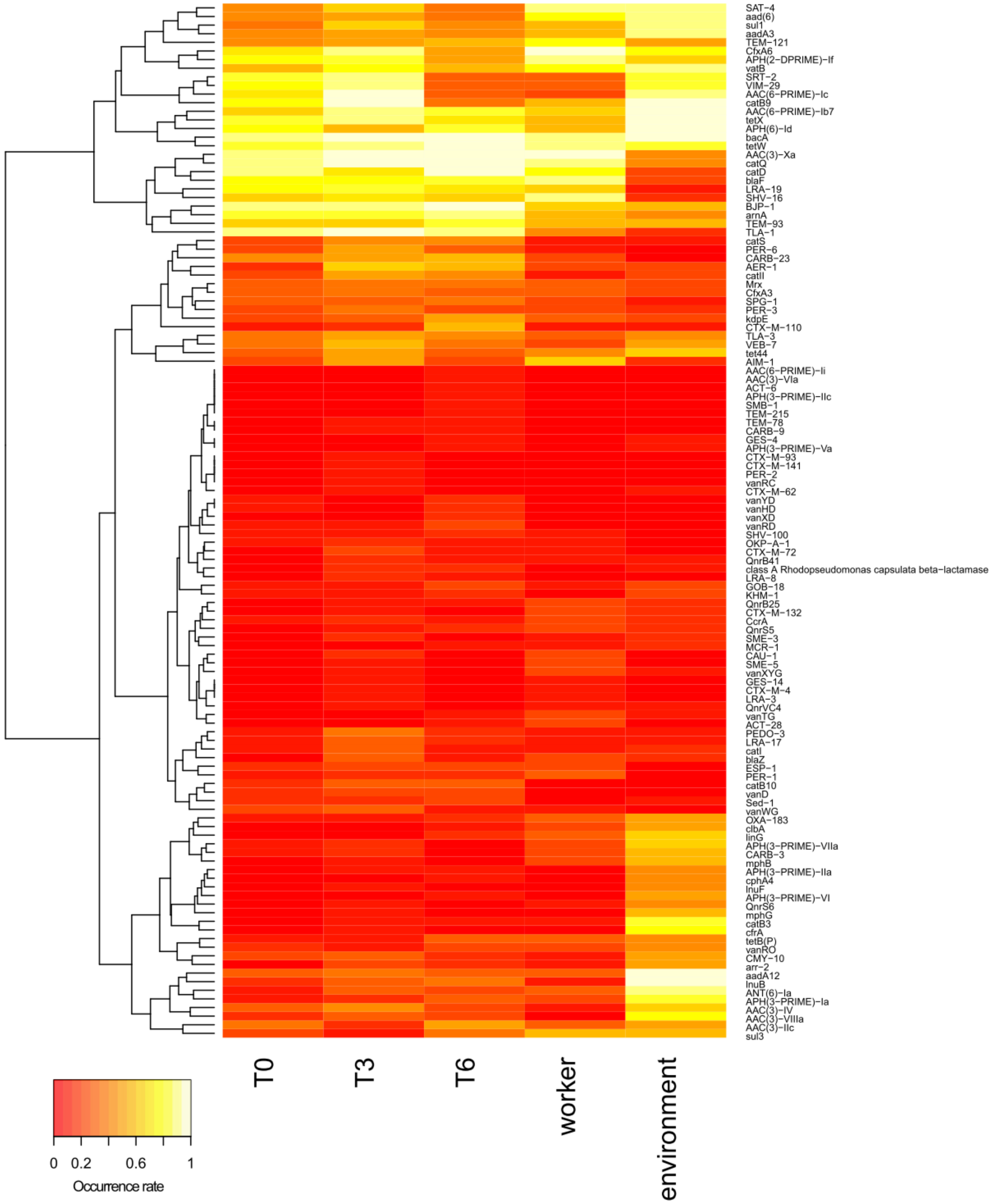

Supplementary Figure 13 | Occurrence of the antibiotic resistance genes in the environmental and human fecal samples. All AR genes that enriched in the students' gut antibiotic resistomes during their stay on swine farm (time point T3) are shown. The occurrence rates of antibiotic resistance genes in each group are represent by color shades in boxes. Source data are provided in the Source Data file.

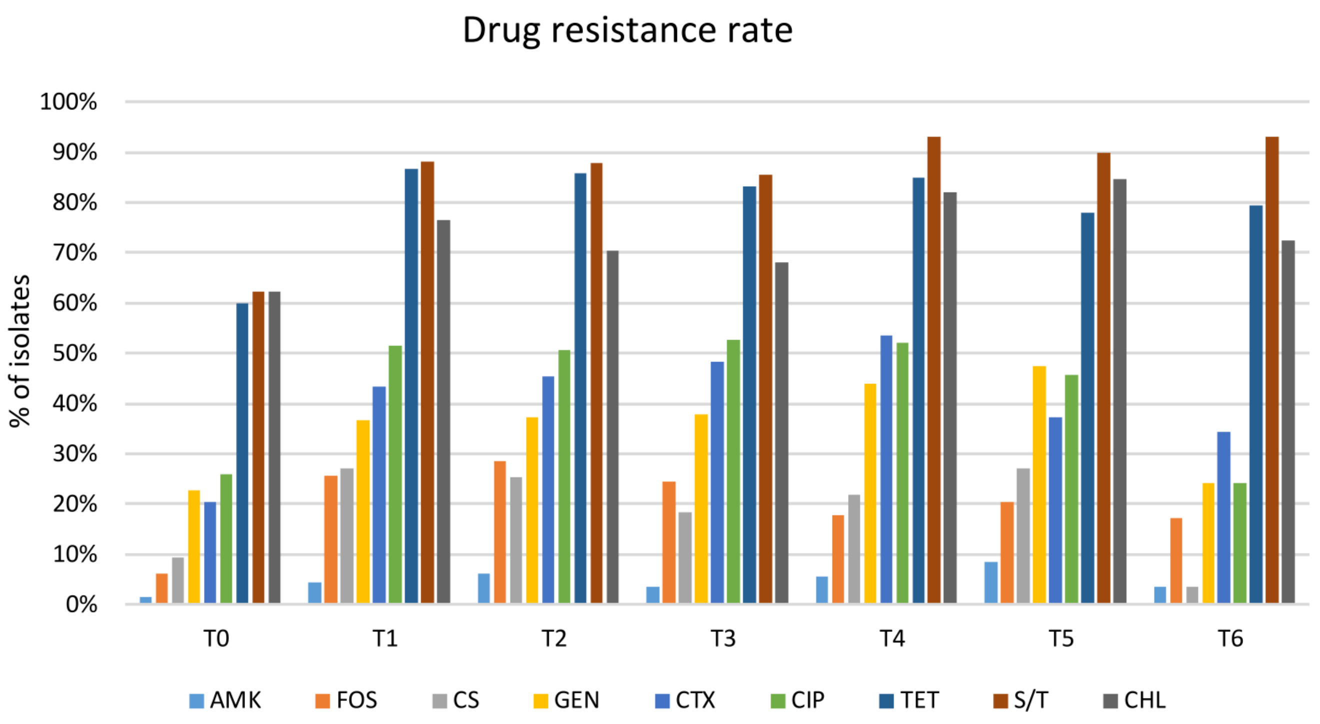

Supplementary Figure 14| Changes in resistance rates of nine antibiotics among isolated *E. coli* strains. Bar plots show the resistance rates of 954 *E. coli* strains isolating from students samples during the students' swine farm before and after residence. Source data are provided in the Source Data file.

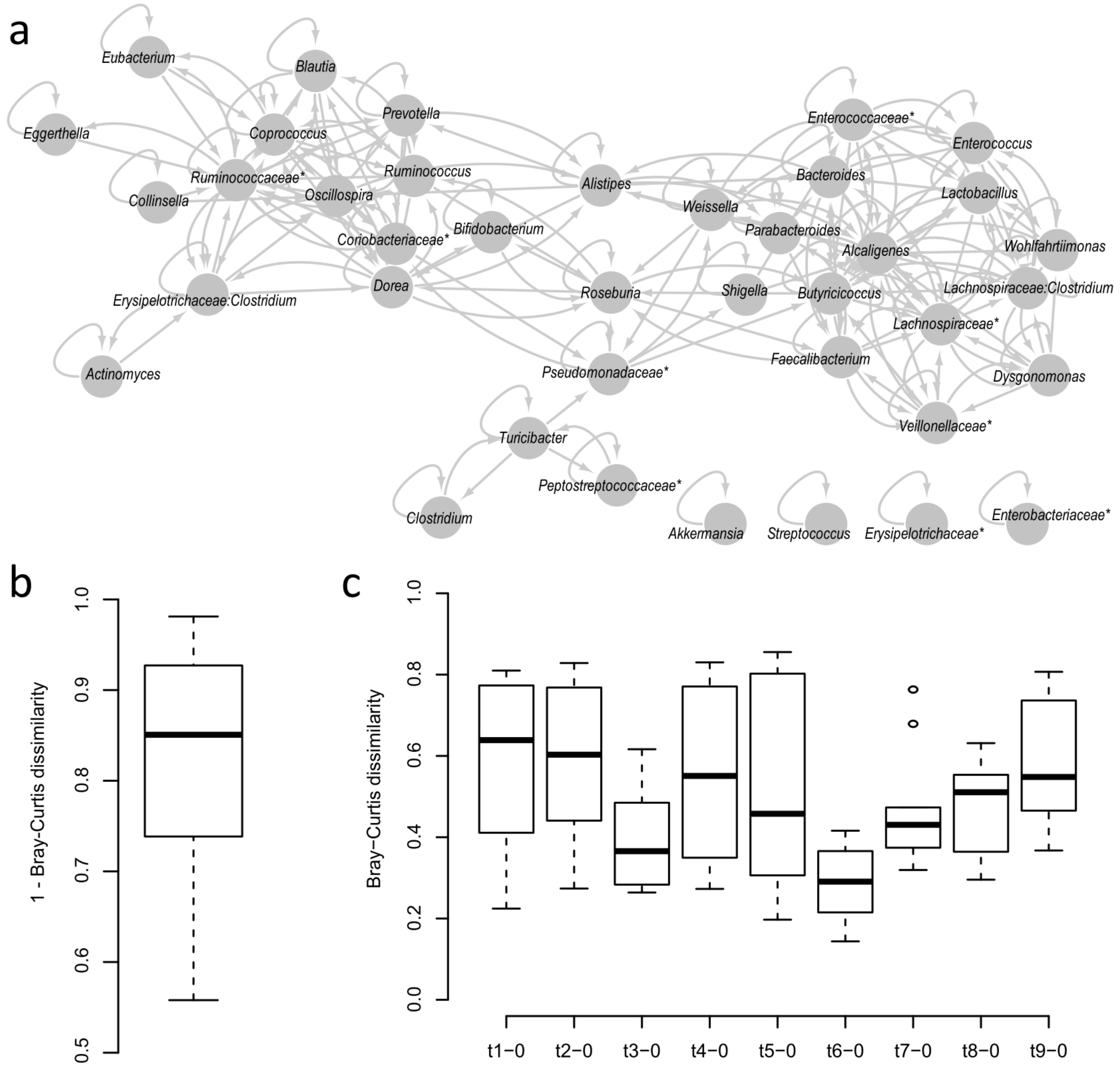

Supplementary Figure 15 | Dynamic Bayesian network of the gut microbiota. (a) Network showing the association between microbial taxa generated by the extended local similarity analysis (eLSA) algorithm. Nodes represent species, and edges represent correlations between two species. (b) Bray-Curtis similarity (1-Bray-Curtis dissimilarity) between the predicted interpolated community structure and the actual community structure of the students' gut microbiota based on leave-one-out cross-validation for the model. (c) Bray-Curtis dissimilarity between T0 and other time points. Boxes show medians/quartiles; error bars extend to the most extreme values within 1.5 interquartile ranges. Source data are provided in the Source Data file.

Reference

1. Qin J*, et al.* A metagenome-wide association study of gut microbiota in type 2 diabetes. *Nature* **490**, 55-60 (2012).

2. Li W, Godzik A. Cd-hit: a fast program for clustering and comparing large sets of protein or nucleotide sequences. *Bioinformatics* **22**, 1658-1659 (2006).

3. Li J*, et al.* An integrated catalog of reference genes in the human gut microbiome. *Nature biotechnology* **32**, 834-841 (2014).

4. Pehrsson EC*, et al.* Interconnected microbiomes and resistomes in low-income human habitats. *Nature* **533**, 212-216 (2016).

5. Pal C, Bengtsson-Palme J, Kristiansson E, Larsson DG. The structure and diversity of human, animal and environmental resistomes. *Microbiome* **4**, 54 (2016).

6. Edelstein M, Pimkin M, Palagin I, Edelstein I, Stratchounski L. Prevalence and molecular epidemiology of CTX-M extended-spectrum beta-lactamase-producing Escherichia coli and Klebsiella pneumoniae in Russian hospitals. *Antimicrob Agents Chemother* **47**, 3724-3732 (2003).

7. Hou J*, et al.* Dissemination of the fosfomycin resistance gene fosA3 with CTX-M beta-lactamase genes and rmtB carried on IncFII plasmids among Escherichia coli isolates from pets in China. *Antimicrob Agents Chemother* **56**, 2135-2138 (2012).
