# Supplementary material for: Environmental remodeling of human gut microbiota and antibiotic resistome in livestock farms
Source: Nat Commun. 2020 Mar 18;11:1427. doi: 10.1038/s41467-020-15222-y (PMC7080799; doi:10.1038/s41467-020-15222-y)
Supplement: Supplementary file 3 — Description of Additional Supplementary Files [file 41467_2020_15222_MOESM3_ESM.docx]

**Description of Supplementary Files**

**File Name: Supplementary Data 1**

**Description:** Information of fecal samples of 14 students in 7 time points and data production using 16S rRNA gene sequencing.

**File Name: Supplementary Data 2**

**Description:** Comparison of the relative abundances of microbial taxa (at the phylum and genus levels) between T0 and other time points.

**File Name: Supplementary Data 3**

**Description:** Data production, de novo assembly and gene prediction of fecal samples of students on time point T0, T3 and T6, and swine farm workers using wholemetagenome shotgun sequencing.

**File Name: Supplementary Data 4**

**Description:** Detailed information of the antibiotic resistance genes identified from human microbiomes.

**File Name: Supplementary Data 5**

**Description:** Data production, de novo assembly and gene prediction of environmental samples of three swine farms using whole-metagenome shotgun sequencing.

**File Name: Supplementary Data 6**

**Description:** Detailed information of the species transmission events.

**File Name: Supplementary Data 7**

**Description:** Detailed information of the draft genomes reconstructed from metagenomics data.

**File Name: Supplementary Data 8**

**Description:** Detailed information of the transmission events of AR genes.
