# Supplementary material for: Environmental remodeling of human gut microbiota and antibiotic resistome in livestock farms
Source: Nat Commun. 2020 Mar 18;11:1427. doi: 10.1038/s41467-020-15222-y (PMC7080799; doi:10.1038/s41467-020-15222-y)
Supplement: Supplementary file 12 — Reporting Summary [file 41467_2020_15222_MOESM12_ESM.pdf]

nature research

Corresponding author(s): Alaric W. D'Souza, Gautam Dantas, Ya-Hong Liu

Last updated by author(s): Feb 13, 2020

Reporting Summary

Nature Research wishes to improve the reproducibility of the work that we publish. This form provides structure for consistency and transparency in reporting. For further information on Nature Research policies, see Authors & Referees and the Editorial Policy Checklist .

Statistics

For all statistical analyses, confirm that the following items are present in the figure legend, table legend, main text, or Methods section.

n/a	Confirmed	
☐	☒	The exact sample size (*n*) for each experimental group/condition, given as a discrete number and unit of measurement
☐	☒	A statement on whether measurements were taken from distinct samples or whether the same sample was measured repeatedly
☐	☒	The statistical test(s) used AND whether they are one- or two-sided<br>*Only common tests should be described solely by name; describe more complex techniques in the Methods section.*
☐	☒	A description of all covariates tested
☐	☒	A description of any assumptions or corrections, such as tests of normality and adjustment for multiple comparisons
☐	☒	A full description of the statistical parameters including central tendency (e.g. means) or other basic estimates (e.g. regression coefficient) AND variation (e.g. standard deviation) or associated estimates of uncertainty (e.g. confidence intervals)
☐	☒	For null hypothesis testing, the test statistic (e.g. *F*, *t*, *r*) with confidence intervals, effect sizes, degrees of freedom and *P* value noted<br>*Give P values as exact values whenever suitable.*
☒	☐	For Bayesian analysis, information on the choice of priors and Markov chain Monte Carlo settings
☒	☐	For hierarchical and complex designs, identification of the appropriate level for tests and full reporting of outcomes
☒	☐	Estimates of effect sizes (e.g. Cohen's *d*, Pearson's *r*), indicating how they were calculated

Our web collection on statistics for biologists contains articles on many of the points above.

Software and code

Policy information about availability of computer code

Data collection	No code was used for data collection.
Data analysis	16S rRNA amplification, sequencing and preprocessing. The V3 and V4 hypervariable regions of the 16S rRNA gene were sequenced and analyzed to define the composition of the bacterial community in human fecal samples. The following amplification primers were used: primer-F = 5′ TCGTCGGCAGCGTCAGATGTGTATAAGAGACAGCCTACGGGNGGCWGCAG; primer-R = 5′ GTCTCGTGGGCTCGGAGATGTGTATAAGAGACAGGAC TACHVGGGTATCTAATCC. For amplicon library preparation, 20 ng of each genomic DNA, 1.25 U Taq DNA polymerase, 5 μl 10× Ex Taq buffer (Mg2+ plus), 10 mM dNTPs (all reagents purchased from TaKaRa Biotechnology Co., Ltd), and 40 pmol of primer mix was used for each 50-μl amplification reaction. For each sample, the 16S rRNA gene was amplified under the following conditions: initial denaturation at 94 °C for 3 min followed by 30 cycles of 94 °C for 45 s, 56 °C for 1 min, and 72 °C for 1 min and a final extension at 72 °C for 10 min. The PCR products were quantified by gel electrophoresis, pooled and purified for reactions. Pyrosequencing was performed on an Illumina MiSeq sequencer with paired-end reads 300 base pairs (bp) in length.<br>Based on the overlaps between the sequenced paired-end reads, the reads were merged into long sequences using the FLASH algorithm (min-overlap = 30, max-overlap = 150)42. Low-quality sequences were then trimmed and eliminated from the analysis based on the following criteria: a) shorter than 400 bp; b) a sequence producing more than 3 'N' bases. Bioinformatic analysis was implemented using the Quantitative Insights into Microbial Ecology QIIME2 platform version 2018.11 (https://qiime2.org/). Briefly, raw Illumina amplicon sequence data were performed quality control process based on DADA2 algorithm, removing the chimeric sequences and truncating the sequences from 5 to 250 bases. Phylogenetic diversity analyses were realized via the q2-phylogeny plugin, which used the mafft45 program to perform multiple sequence alignment on the representative sequences (FeatureData in QIIME2) and the FastTree program to generate phylogenetic tree from the alignments. The microbial community structure (i.e., species richness, evenness and between-sample diversity) of fecal samples was estimated by biodiversity. The Shannon index was used to evaluate alpha diversity, and the weighted and unweighted UniFrac distances were used to evaluate beta diversity. All of these indices were calculated by the QIIME2 pipeline (q2-diversity plugin).<br><br>Metagenomic sequencing and data quality control. The Illumina HiSeq 3000 platform was used to sequence the samples. We constructed a 150-bp paired-end library with an insert size of 350 bp for every sample. The raw sequencing reads for each sample were

independently processed for quality control using the FASTAX Toolkit (http://hannonlab.cshl.edu/fastx_toolkit/). The quality control used the following criteria: (1) reads were removed if they contained more than 3 'N' bases or more than 50 bases with low quality (<Q20); (2) no more than 10 bases with low quality (<Q20) or assigned as N in the tails of reads were trimmed. The remaining reads were then mapped to the human and swine genomes using SOAPaligner2 to remove host DNA contamination. Overall, an average of 0.9% of low-quality or host genome reads was removed from the sequenced samples.

De novo assembly, gene calling and gene catalogue construction. To determine the best assembling method for high-quality whole-metagenome sequencing reads, we compared the performance of two assemblers, SOAPdenovo v2 (previously used in human gut microbiomes) and MEGAHIT v1.1.4 (a de novo assembler for large and complex metagenomic sequences). For SOAPdenovo, we tested the k-mer length ranging from 23 bp to 123 bp by 20-bp steps for each sample and selected the assembled contig set with the longest N50 length. For MEGAHIT parameters "--mink 21 --maxk 119 --step 10 --pre_correction" were used. For most of the samples, MEGAHIT obtained a better assembled contig set than SOAPdenovo; this could be due to its improved assembly of bacterial genomes with highly uneven sequencing depths in metagenomic samples. As a result, we obtained an average of 254.6 ± 72.4 and 754.4 ± 180.4 Mbp (mean ± SD) contig sets for human fecal samples and environmental samples, respectively. The unassembled reads for each ecosystem were pooled and reassembled for further analysis.
Genes were predicted by MetaGeneMark v3.26 based on parameter exploration by the MOCAT pipeline . A non-redundant gene catalogue was constructed using CD-HIT51; from this catalogue, genes with >90% overlap and >95% nucleic acid similarity (no gap allowed) were removed as redundancies. The gene catalogues contained 3,338,109 and 11,374,480 non-redundant genes generated from the human microbiome and the swine farm ecosystem, respectively.

Quantification of metagenomic genes. The abundance of genes in the non-redundant gene catalogues was quantified as the relative abundance of reads. First, the high-quality reads from each sample were aligned against the gene catalogue using SOAP v2.21 using a threshold that allowed at most two mismatches in the initial 32-bp seed sequence and 90% similarity over the whole read. Then, only two types of alignments were accepted: (1) those in which the entirety of a paired-end read could be mapped onto a gene with the correct insert size; (2) those in which one end of the paired-end read could be mapped onto the end of a gene only if the other end of the read mapped outside the genic region. The relative abundance of a given gene in a sample was finally estimated by dividing the number of reads that uniquely mapped to that gene by the length of the gene region and by the total number of reads from the sample that uniquely mapped to any gene in the catalogue. The resulting set of gene relative abundances for all samples was termed a gene profile. The average read mapping rates (or mean reads usage) were 71.5% and 43.8% for human gut microbiome and swine farm environmental samples, respectively.

Quantification of taxa in metagenomic data. We performed the taxonomic profiling (including phylum, class, order, family, genus and species levels) of the metagenomic samples using MetaPhlAn2, which relies on ~1 million clade-specific marker genes derived from 17,000 microbial genomes (including bacterial, archaeal and viral species) to unambiguously classify metagenomic reads to taxonomies and yield relative abundances of taxa identified in the sample.

Alpha diversity (metagenomic data). The Shannon index, calculated as previously described53, was used to represent the within-sample diversity (alpha diversity) of the microbiota in the samples.

Identification and quantification of antibiotic resistance genes: The antibiotic resistance (AR) genes from each metagenomic assemblies were identified by blasting protein sequences against Comprehensive Antibiotic Resistance Database (CARD, downloaded February 2018) database using stringent cutoff (>95%ID and >95 overlap with subject sequence). The remaining unannotated sequences were filtered and subsequently annotated with Resfams core database. This approach resulted in 12,739 unique AR genes from 66 metagenomic assemblies. Together, these 12,739 genes with 2,252 AR sequences from CARD database were used to create high-precision sequence markers using ShortBRED35 (parameters: --clustid 0.95 and --ref Uniref90.fasta).
The ShortBRED results included 20,514 markers for 5,607 AR gene families. The marker list was then manually curated to reduce the rate of false positives in our surveys. Following criteria was used to filter out the false positives:
• genes that confer resistance via overexpression of resistant target alleles (e.g. resistance to antifolate drugs via mutated DHPS and DHFR);
• global gene regulators, two-component system proteins, and signaling mediators;
• efflux pumps that confer resistance to multiple antibiotics;
• genes modifying cell wall charge (e.g. those conferring resistance to polymixins and defensins).
The final set consisted of 1,924 AR gene families. The abundance of AR gene families was measured using shortbred_quantify.py script and about 1,018 AR determinants were detected with RPKM > 0 in at least 2 samples.

Identification of virulence factor genes and antibacterial biocide and metal resistance genes. We identified the virulence factors based on the Virulence Factors of Pathogenic Bacteria Database (VFDB, downloaded February 2018) and the antibacterial biocide and metal resistance genes based on the BacMet database. Amino acid sequences were aligned against the databases using BLASTP (e-value ≤ 1e-5) and assigned to genes by the highest-scoring annotated hit with >80% similarity that covered >70% of the length of the query protein.

Species transmission event identification and SourceTracker. We used a modified SourceTracker algorithm to identify species transmission events from the swine farm environment to human gut microbiota. Briefly, the new genes found in each sample during swine farm residence were grouped into species-level clusters by consistent taxonomic assignment and relative abundance (range: average ±5%). The SourceTracker algorithm was then used to estimate the probability that the species in the fecal sample came from the source environment (probability >80%). The probable transferred species with less than 100 genes or less than 0.01% relative abundance in the human gut microflora were further filtered.
To identify transfer events involving antibiotic resistance genes, SourceTracker was run with the default settings using the environmental microbiota as the source.

Microbial genome reconstruction in metagenomes. We established an approach to reconstruct the genomes of the high-abundance (typically, >3%) species in the human gut metagenomes. Firstly, metagenomic reads were mapped to the closest reference genomes

using SOAP2.21 (>95% identity). The mapped reads were independently assembled using Velvet, an algorithm for de novo short read assembly for single microbial genomes. The software was run multiple times using different k-mer parameters ranging from 39 to 131 to generate the best assembly results. Then, the raw assembled genome was scaffolded by SSPACE v2, and gaps were closed by GapFiller v1.7.0. The short scaffolds were filtered with a minimum length threshold of 200 bp. A circle plot of the draft genomes was obtained using BRIG software. The average nucleotide identity (ANI) between genomes was calculated using the ANIb algorithm, which uses BLAST as the underlying alignment method.

Network visualization. The antibiotic resistance gene co-occurrence network was visualized by Cytoscape 3.3.0 using an edge-weighted spring-embedded layout.

Mobile genetic elements (MGEs). Putative MGE genes, including transposase, integrase, recombinase, phage terminase and endopeptidase genes, and bacterial insertion (IS) sequences were identified from the functional selection by Pfam (v29.0) and KEGG (Kyoto Encyclopedia of Genes and Genomes, downloaded December 2017) annotation. Antibiotic resistance genes were considered to co-localize with an MGE if they shared a contig with an MGE gene in a nearby area (<10 kilobases).

Phylogenetic classification of contigs. Antibiotic resistance contigs and metagenomic assembly contigs were classified using BLASTN with parameters "-word_size 16 -evalue 1e-5 -max_target_seqs 5000" based on the NCBI reference microbial genomes (downloaded December 2017). At least 70% alignment coverage of each contig reads was required. Based on the parameter exploration of sequence similarity across phylogenetic ranks, we used 90% identity as the threshold for species assignment and 85% identity as the threshold for genus assignment.

Creation of the dynamic Bayesian network (DBN) model. The DBN model was created based on genus composition profiles of students' faecal samples at all seven time points. Firstly, we removed 1) two students (H and N) who lacked the sequencing data for at least 2 time points, and 2) the genera with average relative abundance less than 0.5% in students, remaining the gut microbial communities of 12 students on 39 high-abundant genera for further analysis. These genera covered 86% of total relative abundance of analyzed samples. Then, we calculated the genus-genus associations based on the extended local similarity analysis (eLSA) algorithm (default parameters), using the students' genus profiles at all seven time points. The eLSA tool generated an association network from significant associations (permutated $P < 0.01$), including both time-independent (undirected) and time-dependent (directed) associations. For each genus, five most significant associations were remained for simplify the network. Lastly, the partially directed DBN model was created based on the genus-genus association network and the directed associations for each genus from its previous time point to current time point (as shown at Extended Data Fig. 15).

Prediction of the microbial composition based on the DBN model. In the DBN model, the current relative abundance (tn) of every genus can be expressed as a function of the relative abundances of its parent genera at the previous time point (tn-1). The functions in the resulting DBN were derived using Eureqa v1.24.06 (default parameters). Eureqa is a freely downloadable software for deducing equations and hidden mathematical relationships in numerical data sets without prior knowledge of existing patterns. The operations, including constant, add, subtract, multiply, divide, sine, cosine and exponential, were permitted in solutions. Eureqa was allowed to search for best-fitting equations for a maximum of 1 x 1010 formula evaluations, or until correlations >0.8 were observed. To evaluate the accuracy of the DBN model, we trained a new model by using the microbial compositions at time points T0-T5 and then predicted the microbial composition at T6. This leave-one-out cross-validation strategy was also used to predict the compositions of time points T1-T5. For all samples, their predicted microbial compositions achieved high consistency by Bray-Curtis similarity (1-Bray-Curtis distance). Finally, in our dataset, we predicted the relative abundance of all genera at an extrapolated time point (T7) based on the formulas, using their abundances at time point T6. Similarly, the microbial communities at time points T8 and T9 were predicted based on T7 and T8.

Statistical analysis. Statistical analysis was implemented using the R platform. Principal coordinate analysis (PCoA) was performed using the "ape" package based on the UniFrac distances between samples. dbRDA was performed using the "vegan" package v 2.4-2 based on the Bray-Curtis distances on normalized taxa abundance matrices and visualized using the "ggplot2" package. In analyses of PCoA and dbRDA, the top two principal components of the samples were shown, and the Mann–Whitney U-test was used to evaluate the significance of differences in samples obtained at different time points. PERMANOVA was used to determine the significance of time points on the subject's gut microbiota as well as antibiotic resistome. We implemented PERMANOVA using the adonis function based on the Bray-Curtis dissimilarity and 999 permutations. This function calculates the interpoint dissimilarities of each group and compares these values to the interpoint dissimilarities of all points to generate a pseudo-F statistic. This pseudo-F statistic is then compared to the distribution of pseudo-F statistics generated when the function is run on the dissimilarity matrix with permuted labels. Procrustes analysis was performed using the "vegan" package, and the significance of the Procrustes statistic (a correlation-like statistic derived from the symmetric Procrustes sum of squares) was estimated by the protest function with 999 permutations. Rarefaction analysis implemented by in-house Perl scripts was performed to assess the gene richness of environmental samples. Statistical significance was set at $P < 0.05$.

For manuscripts utilizing custom algorithms or software that are central to the research but not yet described in published literature, software must be made available to editors/reviewers. We strongly encourage code deposition in a community repository (e.g. GitHub). See the Nature Research guidelines for submitting code & software for further information.

Data

Policy information about availability of data

All manuscripts must include a data availability statement. This statement should provide the following information, where applicable:

- Accession codes, unique identifiers, or web links for publicly available datasets
- A list of figures that have associated raw data
- A description of any restrictions on data availability

Data Availability and Author Information Assembled functional metagenomic contigs and 16S and shotgun metagenomic reads have been deposited to EBI BioProject (PRJEB20626).

Field-specific reporting

Please select the one below that is the best fit for your research. If you are not sure, read the appropriate sections before making your selection.

☒ Life sciences ☐ Behavioural & social sciences ☐ Ecological, evolutionary & environmental sciences

For a reference copy of the document with all sections, see nature.com/documents/nr-reporting-summary-flat.pdf

Life sciences study design

All studies must disclose on these points even when the disclosure is negative.

Sample size

Study design. Fourteen senior class veterinary students (Student ID: H, I, J, K, L, M, N, O, P, Q, W, X, Y, Z) provided their written informed consent and voluntarily enrolled in the study during participation in an approximately 3-month-long practical training course in veterinary science at South China Agricultural University (SCAU) from July to October 2015. The 14 students were randomly divided into three groups of four to five persons, and each group was assigned to one of three swine farms in three different Chinese provinces, including (from north to south), Henan (Farm ID: H farm), Jiangxi (Farm ID: D farm), and Guangdong (Farm ID: S farm) (Extended Data Fig. 1a). These are typical large-scale swine farms, and all have been in operation for more than 5 years. Three farms implement self-breeding, and all use the closed-end management model. Among them, H farm is the largest, with 15,000 sows, D farm (7,400 sows) is the next largest, and S farm (3,800 sows) is the smallest. Due to limitations in the volunteer veterinary student population, all subjects were male and a parallel group of swine farm unexposed students was not possible. We have taken several steps to mitigate these limitations, including comparisons to a healthy cohort from urban Chinese individuals. To control for differences at individual level, the students' fecal samples were collected longitudinally and the fecal samples at the phase before arriving at the farm (T0) were considered a blank control. In addition, four to five farm workers in each swine farm were also recruited in this study. All the farm workers had engaged in pig farming for 4-18 years and stayed at the present farm at least for one year. The volunteers signed an informed consent form and were asked to agree to fecal swabbing and to complete a short questionnaire related to personal information such as age and gender, personal hygiene, dietary habits, antibiotic use, hospitalization, previous visits to farms or factories, and other pertinent factors (Supplementary Questionnaire; Supplementary Table 1). In addition to environmental exposure, other factors such as diet and work stress may be the important factors influencing the human gut microbiota. Considering that these factors may be caused by environmental changes, in this study, we consider these related factors as environmental impacts.

Sample collection. The students' fecal samples were collected at the following intervals: 1) 1-2 weeks prior to their entry into the swine farm, 2) weekly for the 3 consecutive months of their stay at the swine farm; 3) monthly for another 3 consecutive months after their return to the university. At each swine farm, four to five farm workers who had worked on the farm for at least one year were recruited, and their fecal samples were collected monthly during students' swine farm stays. In addition, averages of 40 pig feces samples, 3 soil samples, 3 sewage samples, and 3 ventilation dust samples for each farm, were collected monthly for the 3 consecutive months of the students stay at the swine farm. Among them, 42 students' fecal samples and 12 pooled samples consisting of 55 environmental samples (around 3-5 samples for each item per farm) from the swine farms, including pig feces, soil, sewage, and ventilation dust, were used in the metagenomic sequencing (Supplementary Table 4; Supplementary Table 6). Samples were submitted using an assigned student study ID and date. Samples were kept on dry ice during transport and were stored at −80 °C prior to DNA extraction and chemical analysis.

Data exclusions

No data was excluded from the analysis.

Replication

This study was conducted simultaneously for 14 students in 3 different farms. We saw similar results from all three farms.

Randomization

The 14 students were randomly divided into three groups of four to five persons, and each group was assigned to one of three swine farms in three different Chinese provinces, including (from north to south), Henan (Farm ID: H farm), Jiangxi (Farm ID: D farm), and Guangdong (Farm ID: S farm) (Extended Data Fig. 1a).

Blinding

Investigators in the study did not have ability to alter group randomization for students. Investigators were not blinded to sample timepoint or StudyID since this information was critical to the analysis.

Reporting for specific materials, systems and methods

We require information from authors about some types of materials, experimental systems and methods used in many studies. Here, indicate whether each material, system or method listed is relevant to your study. If you are not sure if a list item applies to your research, read the appropriate section before selecting a response.

Materials & experimental systems

n/a	Involved in the study	
☒	☐	Antibodies
☒	☐	Eukaryotic cell lines
☒	☐	Palaeontology
☒	☐	Animals and other organisms
☐	☒	Human research participants
☒	☐	Clinical data

Methods

n/a	Involved in the study	
☒	☐	ChIP-seq
☒	☐	Flow cytometry
☒	☐	MRI-based neuroimaging

Human research participants

Policy information about studies involving human research participants

Population characteristics	Fourteen senior class veterinary students (Student ID: H, I, J, K, L, M, N, O, P, Q, W, X, Y, Z) provided their written informed consent and voluntarily enrolled in the study during participation in an approximately 3-month-long practical training course in veterinary science at South China Agricultural University (SCAU) from July to October 2015. The 14 students were randomly divided into three groups of four to five persons, and each group was assigned to one of three swine farms in three different Chinese provinces, including (from north to south), Henan (Farm ID: H farm), Jiangxi (Farm ID: D farm), and Guangdong (Farm ID: S farm). Due to limitations in the volunteer veterinary student population, all subjects were male and a parallel group of swine farm unexposed students was not possible. We have taken several steps to mitigate these limitations, including comparisons to a healthy cohort from urban Chinese individuals. To control for differences at individual level, the students' fecal samples were collected longitudinally and the fecal samples at the phase before arriving at the farm (T0) were considered a blank control. In addition, four to five farm workers in each swine farm were also recruited in this study. All the farm workers had engaged in pig farming for 4-18 years and stayed at the present farm at least for one year. The volunteers signed an informed consent form and were asked to agree to fecal swabbing and to complete a short questionnaire related to personal information such as age and gender, personal hygiene, dietary habits, antibiotic use, hospitalization, previous visits to farms or factories, and other pertinent factors (Supplementary Questionnaire; Supplementary Table 1). In addition to environmental exposure, other factors such as diet and work stress may be the important factors influencing the human gut microbiota. Considering that these factors may be caused by environmental changes, in this study, we consider these related factors as environmental impacts.
Recruitment	Students voluntarily enrolled in the study during participation in an approximately 3-month-long practical training course in veterinary science at South China Agricultural University (SCAU) from July to October 2015. Due to limitations in the volunteer veterinary student population, all subjects were male and a parallel group of swine farm unexposed students was not possible. We have taken several steps to mitigate these limitations, including comparisons to a healthy cohort from urban Chinese individuals. To control for differences at individual level, the students' fecal samples were collected longitudinally and the fecal samples at the phase before arriving at the farm (T0) were considered a blank control. In addition, four to five farm workers in each swine farm were also recruited in this study. All the farm workers had engaged in pig farming for 4-18 years and stayed at the present farm at least for one year. Due to these recruitment limitations results could be different for females traveling or staying on swine farms.
Ethics oversight	The Institutional Review Board of South China Agricultural University (SCAU-IRB) approved the protocols. All animals were sampled under authorization from Animal Research Committees of South China Agricultural University (SCAU-IACUC).

Note that full information on the approval of the study protocol must also be provided in the manuscript.
